# Haematological Predictors of Cirrhosis in Paediatric Wilson Disease: A Record-Based Analysis

**DOI:** 10.7759/cureus.94806

**Published:** 2025-10-17

**Authors:** Sukru Gungor, Fatma İ Varol, Yurday Öncül, Arzu Akyay, Mukadder A Selimoğlu, Emre Gök

**Affiliations:** 1 Paediatric Gastroenterology, Hepatology and Nutrition, Inonu University, Malatya, TUR; 2 Paediatric Hematology and Oncology, Inonu University, Malatya, TUR; 3 Paediatric Hematology and Oncology, Faculty of Medicine, Inonu University, Malatya, TUR

**Keywords:** cirrhosis, database, haematology, paediatric, wilson disease

## Abstract

Background

The study aimed to explore the prognostic value of haematological parameters in Wilson disease (WD) patients.

Methods

Laboratory data at the time of diagnosis of 136 patients diagnosed with WD at the Paediatric Hepatology Clinic of İnönü University Faculty of Medicine between 2010 and 2022 were retrospectively analysed. A total of 81 patients with available histopathological data were grouped according to the presence of cirrhosis.

Results

Retrospective analysis of laboratory data of WD patients revealed significant correlations. While there was a positive correlation between the Mentzer index, red blood cell distribution width (RDW), mean corpuscular volume (MCV), and mortality scores, haemoglobin (Hb), red blood cell (RBC), platelet (PLT), and plateletcrit (PCT) showed a significant negative correlation. In the haematological data (PLT, RDW, PCT), important cut-off points were identified with high sensitivity and specificity that could predict cirrhosis. When we analyse the risk for cirrhosis according to these cut-off points, we found that, if the Mentzer index is >21.47, the risk increases approximately 11 times; if PLT ≤158, the risk increases 6.6 times; if MCV >85.3, the risk increases approximately five times; if RBC ≤3,980 x 10^3^ UL, the risk increases approximately 13 times; if PCT ≤0. 22, the risk increased approximately 12-fold; if RDW ≥14.7, the risk increased approximately 12-fold; if Hb/RDW ratio ≤0.83, the risk increased 10-fold; and if AAR ≥0.73, the risk increased eightfold.

Conclusion

The findings suggest that haematological parameters can be valuable for clinicians in determining the prognosis of WD patients.

## Introduction

Wilson disease (WD) is a multisystem disorder in which copper accumulates in various tissues, exceeding the carrying capacity of ceruloplasmin and causing tissue damage, as a result of mutations in the ATP7B gene found in many tissues. Although the clinical manifestations of WD are often hepatic and neuropsychiatric, it can also present as renal (renal tubular acidosis, tubular dysfunction, nephrolithiasis), cardiac (arrhythmias, cardiomyopathy), cutaneous (lipoma, azure lunulae in nails, anetoderma, xerosis, acanthosis nigricans), osteoarticular (osteodystrophies), endocrinological (hypothyroidism, hypoparathyroidism, pubertal disorders, infertility or recurrent miscarriages), ocular (Kayser-Fleischer ring, sunflower cataract), haematological (Coombs-negative haemolytic anaemia), and other organ disorders [1]. Haematological findings in WD are not limited to haemolysis. Cirrhosis and portal hypertension resulting from chronic liver damage also cause severe haematological abnormalities, including leukopenia and thrombocytopenia due to coagulopathy and hypersplenism [2].

Reliable tools for predicting survival and mortality in acute liver failure are not fully developed. Laboratory findings (total bilirubin, international normalised ratio (INR), phosphorus, lactate, prothrombin time (PT), ammonia), clinical features (encephalopathy, nutritional status, cerebral oedema), diagnosis, aetiological factors (paracetamol intoxication and others), and/or efforts are being made to develop prognostic measurement tools that incorporate all of these [3].

Although it has been emphasised that haemolytic anaemia and/or low haemoglobin may be a marker in the diagnosis of acute liver failure [4], the effects of other haematological parameters on diagnosis and prognosis are still among the issues that need to be investigated. We believe that haematological parameters may be a cheap, easy-to-measure, and readily available method to determine prognosis and cirrhosis in WD patients. Therefore, in our study, we aimed to investigate the effects of haematological data on prognosis in WD.

## Materials and methods

Laboratory data of 136 patients diagnosed with WD at the Paediatric Hepatology Unit of İnönü University Faculty of Medicine, Malatya, Turkey, between 2010 and 2022 were retrospectively reviewed. Of these, complete haematological and histopathological data were available for 81 patients, who were therefore included in the statistical analysis, while 55 patients were excluded due to incomplete data.

This study was approved by the Local Ethics Committee of the Faculty of Medicine of a local tertiary care university hospital (Decision Date: 05-09-2023, Decision no: 2023/4896, Decision: Positive). The study was conducted in accordance with the principles of the Declaration of Helsinki.

Diagnosis of WD

According to the Leipzig scoring system, those with a score ≥4 were considered to have WD [5].

Groups

Patients were grouped according to the presence or absence of cirrhosis. The diagnosis of cirrhosis was made based on the histopathological evaluation of liver biopsy specimens according to the Metavir fibrosis scoring system (F0-F4) [6].

According to the Metavir system, F0 indicates no fibrosis; F1, portal fibrosis without septa; F2, portal fibrosis with few septa; F3, portal fibrosis with multiple septa; and F4 was defined as cirrhosis.

Inclusion criteria

Patients aged between 3 and 18 years with a diagnosis of WD whose haematological, biochemical, and histopathological data included Metavir fibrous score data were included in the study.

Exclusion criteria

Among these, patients whose haematological, biochemical, or histopathological data were incomplete (n = 55), or whose histopathological records lacked Metavir fibrosis scoring, were excluded from the final analysis. In addition, patients older than 18 years and those without confirmed WD were excluded.

Hematological parameters

Haematological parameters of all patients included in the study (white blood cell (WBC), neutrophil, lymphocyte, basophil, eosinophil, monocyte, haemoglobin (Hb), red blood cell (RBC), platelet (PLT), platelet large cell ratio (PLCR), mean corpuscular volume (MCV), mean platelet volume (MPV), red blood cell distribution width (RDW), platelet distribution width (PDW), plateletcrit (PCT), granulocyte index (IG), granulocyte percentage (IG%), partial thromboplastin time (PTT), prothrombin time (PT), international normalized ratio (INR), lymphocyte-monocyte ratio (LMO), neutrophil-lymphocyte ratio (NLR), Hb/RDW ratio, gamma-glutamyl transferase to platelet ratio (GPR), Mentzer index, paediatric end-stage liver disease (PELD), model for end-stage liver disease (MELD), aspartate-alanine aminotransferase ratio (AAR), aspartate aminotransferase to platelet ratio index (APRI), and fibrosis-4 index (FIB4)) were analysed retrospectively.

A total of 81 patients whose complete haematological, biochemical, and histopathological data were available were included in the final analysis. Only data obtained from the same laboratory instruments and standardized analytic units at the Paediatric Hepatology Unit of İnönü University Faculty of Medicine were included. Patients whose laboratory results were measured using non-standardised techniques or different analysers were excluded to ensure methodological consistency throughout the 12-year study period.

Indices

The following were the indices used [7,8].

Mentzer Index: \[ \text{Mentzer Index} = \frac{\text{MCV}}{\text{RBC}} \]

Aspartate aminotransferase to PLT ratio index (APRI) = \[ \text{APRI} = \left( \frac{\text{AST (U/L)}}{\text{ULN}} \; \bigg/ \; \text{PLT} \; ( \times 10^{9}/\text{L}) \right) \times 100 \]

Fibrosis-4 index (FIB4) = \[ \text{FIB-4} = \frac{\text{Age (years)} \times \text{AST (IU/L)}}{\text{PLT} \; ( \times 10^{9}/\text{L}) \times \sqrt{\text{ALT (U/L)}}} \]

Proportions:

LMO = \[ \text{LMO} = \frac{\text{Lymphocyte}}{\text{Monocyte}} \]

NLR = \[ \text{NLR} = \frac{\text{Neutrophil}}{\text{Lymphocyte}} \]

AAR: \[ \text{AAR} = \frac{\text{AST}}{\text{ALT}} \]

GPR: \[ \text{GPR} = \frac{\text{GGT (IU/L)}}{\text{PLT} \; ( \times 10^{9}/\text{L})} \]

Hb/RDW Ratio: \[ \text{Hb/RDW Ratio} = \frac{\text{Hb (g/dL)}}{\text{RDW (\%)}} \]

Prognostic scores

Model for end-stage liver disease (MELD) and paediatric end-stage liver disease (PELD): The PELD score was used for all patients aged <12 years, and the MELD score for all patients aged ≥12 years. Using data, including albumin, bilirubin, INR, growth parameters, creatinine, PELD scores, and MELD scores, were calculated based on online application data (https://www.mdcalc.com) [9].

Statistical analysis

The Statistical Product and Service Solutions (SPSS, version 22; IBM SPSS Statistics for Windows, Armonk, NY) was used. Variables were expressed as mean ± standard deviation, number (n), and percentage (%). The Kolmogorov-Smirnov test was used to determine whether numerical variables were normally distributed. Student's t-test or one-way analysis of variance (ANOVA) was used for normally distributed parameters, and Mann-Whitney U-test or Kruskal-Wallis test for non-normally distributed parameters. Chi-squared test, Student's t-test, or the Mann-Whitney U-test were used to test the statistical significance. An independent Student's t-test was used to determine the arithmetic mean of a dependent variable and whether there was a significant difference between two independent groups. A one-way ANOVA test was used to determine the arithmetic mean of a dependent variable in more than two independent groups and whether there was a significant difference between groups. ROC curve analysis was performed to determine diagnostic cut-off points in haematological measurements that could predict cirrhosis. Risk analysis was performed with a logistic regression test for the risk of developing cirrhosis on haematological parameters, whose cut-off point was determined by ROC curve analysis. Correlation analysis was used to determine whether there was a linear relationship between two numerical variables and, if so, the direction and strength of this relationship. Pearson's correlation was preferred because the data were normally distributed. A value of p < 0.05 was considered statistically significant.

## Results

Haematological data from 81 patients with WD were retrospectively reviewed. The records showed that 20 patients (14.7%) had haemolytic anaemia. There were 81 out of 136 patients (59%) with histopathological findings in the liver. The mean age of these patients was 9.52 ± 3.76 (3.5-17.9) years. A total of 49 (60.5%) patients were male. Histopathological evidence of cirrhosis was found in 51 (63%) of these patients.

When haematological parameters of WD patients with and without cirrhosis were evaluated, Hb, RBC, PLT, PCT, and Hb/RDW ratio values were found to be statistically significantly lower in the cirrhosis group (p < 0.001) (Table 1). MCV, RDW, INR, Hb/RDW, Mentzer index, PELD, MELD, and AAR values were statistically significantly higher in patients with cirrhosis. No statistically significant differences were observed between the cirrhotic and non-cirrhotic groups regarding age and sex distribution (Table 1).

**Table 1:** Evaluation of hematological parameters of Wilson disease (WD) with and without cirrhosis Statistics: Independent Student's t-test. The data are presented as N (%), mean ± standard deviation (Mean±SD). A p-value of less than 0.05 was considered statistically significant. Abbreviations: WD: Wilson desease, WBC: White blood cell, Hb: Hemoglobin, RBC: Red blood cell, PLT: Platelet, PLCR: Platelet large cell ratio, MCV: Mean corpuscular volume, RDW: Red blood cell distribution volume, PCT: Plateletcrit, IG: Granulocyte index, IG%: Percentage of granulocytes, PTT: Partial thromboplastin time, PT: Prothrombin time, INR: International normalized ratio, LMO: Lymphocyte-monocyte ratio, NLR: Neutrophil-lymphocyte ratio, GPR: Gamma glutamyl transferase-platelet ratio, PELD: Paediatric end-stage liver disease, MELD: End-stage liver disease model, AAR: aspartate-alanine aminotransferase ratio, APRI: Aspartate aminotransferase to platelet ratio index, FIB4: Fibrosis-4 index

Parameter	WD without cirrhosis (n=30, Mean ± SD)	WD with cirrhosis (n=51, Mean ± SD)	t value/ χ² value	p value
	n-%	n-%		
Sex				
Female	11-36.7	21-41.2	0.161	0.688
Male	19-63.3	30-5.8		
Age	9.03 ± 4.3	9.8 ± 3.41	-0.887	0.378
WBC (×10³/µL)	7.305 ± 2.933	7.021 ± 4.179	0.328	0.744
Neutrophil (×10³/µL)	3.434 ± 2.172	3.397 ± 2.758	0.062	0.950
Lymphocyte (×10³/µL)	2.730 ± 1.423	2.045 ± 1.379	2.131	0.036
Basophil (×10³/µL)	0.086 ± 0.244	0.082 ± 0.205	0.064	0.949
Eosinophil (×10³/µL)	0.308 ± 0.349	0.238 ± 0.264	1.018	0.312
Monocyte (×10³/µL)	0.560 ± 0.246	0.626 ± 0.390	-0.927	0.357
Hb (g/dL)	12.85 ± 1.59	10.80 ± 2.63	4.342	<0.001
RBC (×10⁶/µL)	4.772 ± 0.575	3.764 ± 1.119	5.339	<0.001
PLT (×10⁹/L)	320.99 ± 159.16	174.35 ± 111.93	4.441	<0.001
PLCR (%)	30.35 ± 10.75	31.20 ± 7.03	-0.278	0.783
MCV (fL)	80.72 ± 7.11	88.12 ± 10.41	-3.447	<0.001
MPV (fL)	9.32 ± 1.77	10.98 ± 11.08	-0.814	0.418
RDW (%)	14.81 ± 3.69	17.56 ± 3.86	-3.135	0.002
PDW (%)	14.55 ± 3.18	14.89 ± 3.42	-0.450	0.654
PCT (%)	0.299 ± 0.14	0.16 ± 0.11	5.028	<0.001
IG (×10³/µL)	0.066 ± 0.113	0.083 ± 0.195	-0.277	0.784
IG (%)	0.378 ± 0.410	0.615 ± 1.370	-0.580	0.567
PTT (sec)	32.64 ± 7.72	39.13 ± 12.48	-2.567	0.012
PT (sec)	21.37 ± 39.63	1.89 ± 1.21	-0.662	0.510
INR	1.21 ± 0.43	1.89 ± 1.21	-3.616	<0.001
LMO	5.26 ± 3.13	3.78 ± 2.15	2.512	0.014
NLR	1.92 ± 2.28	2.37 ± 2.52	-0.808	0.421
Hb/RDW ratio	0.910 ± 0.221	0.657 ± 0.247	4.613	<0.001
GPR	0.0005 ± 0.0012	0.009 ± 0.0009	-1.822	0.072
Mentzer Index	17.27 ± 3.44	27.37 ± 16.09	-4.318	<0.001
PELD	3.62 ± 8.37	18.97 ± 19.22	-4.027	<0.001
MELD	7.64 ± 2.73	25.00 ± 12.47	-7.199	<0.001
AAR	1.14 ± 1.46	2.29 ± 2.00	-2.713	0.008
APRI	0.334 ± 1.323	0.219 ± 0.332	0.570	0.570
FIB-4	1.96 ± 10.24	0.40 ± 1.21	0.831	0.413

When evaluating the correlation between haematological parameters and mortality scores, a significant positive correlation was found between Metavir fibre score and INR, Mentzer index, MCV, and RDW, and a significant negative correlation was found between Hb, PLT, RBC, PCT, HbRDW, and LMO (Table 2).

**Table 2:** Evaluation of the correlation between laboratory data and mortality and other scoring in patients with Wilson disease Statistics: Pearson corelation. The data are presented as N (%), mean ± standard deviation (Mean±SD). A p-value of less than 0.05 was considered statistically significant. Abbreviations: WD: Wilson desease, WBC: White blood cell, Hb: Hemoglobin, RBC: Red blood cell, PLT: Platelet, PLCR: Platelet large cell ratio, MCV: Mean corpuscular volume, RDW: Red blood cell distribution volume, PCT: Plateletcrit, IG: Granulocyte index, IG%: Percentage of granulocytes, PTT: Partial thromboplastin time, PT: Prothrombin time, INR: International normalized ratio, LMO: Lymphocyte-monocyte ratio, NLR: Neutrophil-lymphocyte ratio, GPR: Gamma glutamyl transferase-platelet ratio, PELD: Paediatric end-stage liver disease, MELD: End-stage liver disease model, AAR: aspartate-alanine aminotransferase ratio, APRI: Aspartate aminotransferase to platelet ratio index, FIB4: Fibrosis-4 index

Variables		Metavir Fibrosis Score	MELD Score	PELD Score	AAR	APRI	FIB4
WBC	Pearson Correlation	0.096	0.389^**^	0.285^*^	0.193	-0.192	-0.169
	Sig. (2-tailed)	0.396	0.008	0.037	0.092	0.095	0.142
INR	Pearson Correlation	0.278^*^	0.657^**^	0.595^**^	0.617^**^	0.054	-0.019
	Sig. (2-tailed)	0.012	0.000	0,000	0.000	0.638	0.869
Hb	Pearson Correlation	-0.340^**^	-0.703^**^	-0.609^**^	-0.525^**^	-0.005	0.061
	Sig. (2-tailed)	0.002	0.000	0.000	0.000	0.969	0.596
Mentzer İndeksi	Pearson Correlation	0.305^**^	0.646^**^	0.600^**^	0.531^**^	0.030	-0.024
	Sig. (2-tailed)	0.006	0.000	0,000	0.000	0.794	0.837
PLT	Pearson Correlation	-0.326^**^	-0.337^*^	-0,371^**^	-0.400^**^	-0.249^*^	-0.156
	Sig. (2-tailed)	0.003	0.022	0.006	0.000	0.029	0.176
MCV	Pearson Correlation	0.294^**^	0.596^**^	0.490^**^	0.485^**^	0.107	0.059
	Sig. (2-tailed)	0.008	0.000	0.000	0.000	0.352	0.613
RBC	Pearson Correlation	-0.389^**^	-0.821^**^	-0.712^**^	-0.619^**^	-0.070	0.013
	Sig. (2-tailed)	0.000	0.000	0.000	0.000	0.548	0.914
PCT	Pearson Correlation	-0.312^**^	-0.416^**^	-0.461^**^	-0.403^**^	-0.245^*^	-0.161
	Sig. (2-tailed)	0.005	0.004	0.000	0.000	0.032	0.162
RDW	Pearson Correlation	0.262^*^	0.670^**^	0.620^**^	0.331^**^	-0.049	-0.088
	Sig. (2-tailed)	0.019	0.000	0.000	0.003	0.677	0.451
HbRDW ratio	Pearson Correlation	-0.374^**^	-0.766^**^	-0.656^**^	-0.501^**^	0.010	0.084
	Sig. (2-tailed)	0.001	0.000	0.000	0.000	0.929	0.473
MPV	Pearson Correlation	0.088	-0.040	-0.495^**^	-0.008	-0.014	-0.011
	Sig. (2-tailed)	0.437	0.790	0.000	0.942	0.907	0.926
RDWPLT ratio	Pearson Correlation	0.000	0.431^**^	0.270	0.580^**^	0.224	0.068
	Sig. (2-tailed)	0.997	0.003	0.051	0.000	0.051	0.557
RDWLenfosit ratio	Pearson Correlation	-0.024	0.160	0.160	0.302^**^	0.261^*^	0.174
	Sig. (2-tailed)	0.830	0.294	0.254	0.008	0.023	0.133
PDW	Pearson Correlation	0.065	0.066	0.544^**^	0.074	0.158	0.131
	Sig. (2-tailed)	0.569	0.667	0.000	0.524	0.173	0.259
IG	Pearson Correlation	0.056	0.080	0.499^*^	0.233	0.086	0.085
	Sig. (2-tailed)	0.763	0.804	0.049	0.215	0.653	0.655
IG%	Pearson Correlation	0.054	0.419	0.538^*^	0.384^*^	0.091	-0.048
	Sig. (2-tailed)	0.768	0.175	0.032	0.036	0.633	0.802
LMO	Pearson Correlation	-0.247^*^	-0.284	-0.329^*^	-0.299^**^	-0.140	-0.093
	Sig. (2-tailed)	0.026	0.055	0.015	0.008	0.224	0.421
NLR	Pearson Correlation	0.045	0.306^*^	0.174	0.369^**^	0.116	0.036
	Sig. (2-tailed)	0.690	0.039	0.208	0.001	0.314	0.754
GPR	Pearson Correlation	0.104	0.253	0.175	0.488^**^	0.152	-0.013
	Sig. (2-tailed)	0.357	0.090	0.207	0.000	0.188	0.910

We found a positive correlation between MELD score and WBC, INR, Mentzer index, MCV, RDW, RDW/PLT, and NLR, and a significant negative correlation with Hb, PLT, RBC, PCT, Hb/RDW, and LMO. A positive correlation was found between PELD score and WBC, INR, Mentzer index, MCV, RDW, PDW, IG, and IG%, and a significant negative correlation was found between Hb, PLT, RBC, PCT, Hb/RDW, MPV, and LMO.

A significant positive correlation was found between AAR and INR, Mentzer index, MCV, RDW, RDW/PLT, RDW/lymphocyte ratio, IG%, NLR, and GPR, and a negative correlation was found between Hb, PLT, RBC, PCT, Hb/RDW, and LMO.

While there was a weak positive correlation between APRI score and RDW/lymphocyte ratio, a significant weak negative correlation was found between PLT and PCT.

ROC curve analysis was performed to determine the best cut-off points of haematological parameters that could predict the development of cirrhosis. Accordingly, we found that in WD patients, a Mentzer index value ≥21.47 can predict cirrhosis with 55% sensitivity and 93% specificity (p < 0.001) (Table 3).

**Table 3:** Risk analysis for cirrhosis in pediatric WD patients Statistics: Binary Logistic regression analysis. The data are presented as N (%), mean ± standard deviation (Mean±SD). A p-value of less than 0.05 was considered statistically significant. Abbreviations: PLT: platelet, MCV: mean corpuscular volüme, RBC: red blood cell, PCT: Plateletcrit, RDW: Red blood cell distribution volüme, AAR: aspartate-alanine aminotransferase ratio.

Risk factors	OD	95% CI		p value
		Lower	Upper	
Mentzer index (>21.47)	10.96	2.944	40.775	<0.001
PLT (≤158)	6.60	2.175	19.971	<0.001
MCV (>85.3)	4.70	1.704	12.930	0.003
RBC (x10^3^UL) (≤3980)	12.86	3.446	47.972	<0.001
PCT (≤0.22)	11.95	4.068	35.096	<0.001
RDW (≥14.7)	11.95	4.068	35.096	<0.001
Hb/RDWratio (≤0.83)	10.00	3.503	28.543	<0.001
AAR (≥0.73)	8.06	2.821	23.046	<0.001

When the predictive properties of haematological parameters and mortality scores were evaluated, we found that PCT, RDW, Hb/RDW ratio, AAR, MELD score, and PELD values, which are the best cut-off values to predict cirrhosis, had high sensitivity and specificity in predicting cirrhosis (p < 0.001) (Table 4).

**Table 4:** Determination of the best cut-off point of hematological data that can predict cirrhosis in WD patients Statistics: ROC curve analysis. The data are presented as N (%), mean ± standard deviation (Mean±SD). A p-value of less than 0.05 was considered statistically significant. Abbreviations: PLT: Platelet, MCV: Mean corpuscular volume, RBC: Red blood cell, PCT: Plateletcrit, RDW: Red blood cell distribution volume, AAR: Aspartate-alanine aminotransferase ratio, PELD: Paediatric end-stage liver disease, MELD: End-stage liver disease model

Variables	Best cut-off point	Area	Sensitivity	Specificity	PPV	NPV	%95 C.I.	P
Mentzer index	>21.47	0.778	0.549	0.933	0.933	0.549	0.678-0.878	<0.001
PLT (x10^6 ^L)	≤158	0.785	0.568	0.833	0.853	0.53.2	0.680-0.868	<0.001
MCV	>85.3	0.631	0.588	0.800	0.833	0.533	0.604-0.810	<0.001
RBC (x10^3 ^UL)	≤3980	0.775	0.588	0.900	0.909	0.562	0.669-0.861	<0.001
PCT	≤0.22	0.805	0.784	0.766	0.851	0.676	0.701-0.884	<0.001
RDW	>14.7	0.796	0.800	0.800	0.807	0.706	0.691-0.878	<0.001
Hb/RDW ratio	≤0.83	0.780	0.780	0.767	0.848	0.676	0.673-0.865	<0.001
AAR	>0.73	0.792	0.915	0.633	0.796	0.826	0.685-0.876	<0.001
MELD	>8	0.933	0.931	0.823	0.900	0.875	0.818-0.985	<0.001
PELD	>10	0.732	0.636	0.905	0.913	0.613	0.594-0.843	<0.001

When we perform risk analysis for cirrhosis with logistic regression test according to the best cut-off points of haematological parameters that can predict cirrhosis, we found that, if the Mentzer index value is >21.47, the risk increases approximately 11 times; if the PLT value is ≤158 x 10^6 ^L, the risk increases 6.6 times; if the MCV value is >85.3, the risk increases approximately five times; if the RBC value is ≤3,980 x 10^3 ^UL, the risk increases approximately 13 times; if the PCT value is ≤0.22, the risk increases approximately 12 times; if the RDW value is ≥14.7, the risk increases approximately 12 times; if the Hb/RDW ratio is ≤0.83, the risk increases 10-fold; and if the AAR value is ≥0.73, the risk increases eight times.

## Discussion

Hemolytic anaemia is a common haematological finding in WD. It occurs with a frequency of 10-15% [2,10]. The characteristic of this haemolytic anaemia is that it is Coombs-negative and occurs in attacks independent of liver failure [2,11]. In our study, in agreement with the literature, 20 of 136 Wilson patients (14.7%) had Coombs-negative haemolytic anaemia. Fourteen of these patients (70%) had acute fulminant liver failure. This supports case series and studies indicating that it is an indicator of poor prognosis in Wilson patients [4,12].

Some studies have emphasised the prognostic importance of peripheral blood parameters in patients with cirrhosis [13]. It has been reported that RDW can discriminate cirrhosis in chronic hepatitis B patients [14], and mortality is higher in those with RDW greater than 15.1% [15]. Our study supports the literature, and we found that RDW was significantly higher in cirrhotic Wilson patients (Table 1). In addition to the literature, we found that RDW showed a significant positive correlation with mortality scores such as MELD, PELD, and Metavir fibrosis score (Table 2). In our study, we also found that, if the RDW is ≥14.7, the risk of developing cirrhosis increases approximately 12-fold.

It is emphasised in the literature that proportional parameters related to RDW can be prognostic indicators in liver diseases [13]. The Hb/RDW ratio has been found to predict survival in hepatitis B patients [16]. The RDW/PLT ratio has been found to predict liver fibrosis in hepatitis B [17], and the RDW/Lymphocyte ratio can also predict the incidence of cirrhosis in hepatitis B [9]. In our study, the Hb/RDW ratio was higher in the cirrhotic group, which is consistent with the literature. In addition, there was a significant negative correlation with prognostic markers such as AAR, MELD, PELD, and Metavir fibrous score. We found that, when the Hb/RDW ratio is ≤0.83, it can predict cirrhosis with 78% sensitivity and 77% specificity. We found that the risk of cirrhosis increased 10-fold with this cut-off value. These results support the literature. However, in contrast to the literature, there was no significant difference in RDW/PLT and RDW/lymphocyte values between Wilson patients with and without cirrhosis. We believe that this difference with the literature is due to the different patient cohort.

We could not find any study in the literature that investigated the effect of haematological parameters on prognostic markers in Wilson patients. However, Huang et al. found that MCV was significantly lower in patients with alcoholic fatty liver and alcoholic hepatitis than in the cirrhotic group [18]. Another study reported that the increase in MCV value was associated with the severity of decompensated liver failure due to hepatitis B [19]. In addition, Chen et al. [13] reported that RDW and MCV are important indices in predicting cirrhosis in Wilson patients. Mangal et al. also found that there is a statistically significant positive relationship between MCV and Child-Pugh score and that high MCV is associated with the severity of alcoholic liver disease [20]. The patient group in our study is different from that in the literature. However, the data that we obtained support the literature.

In their study comparing autoimmune liver disease and healthy group data, Ustaoglu et al. [21] emphasized that RDW and PDW have significant sensitivity and specificity in determining autoimmune liver disease. If RDW >13.7, its sensitivity was 76% and specificity was 62%, respectively (AUC: 0.74; p < 0.001). If PDW was >17.9%, the sensitivity was 80%, and the specificity was 71% (AUC: 0.85; p < 0.001). In our study, the sensitivity and specificity of RDW in predicting cirrhosis in Wilson patients were 80%. This finding was consistent with the literature. However, the PDW value was not different in Wilson patients with and without cirrhosis in our study. This may be due to the differences in the patient groups included in the study.

Cirrhosis in patients with WD is an important factor affecting patient management, quality of life, and prognosis. Prognostic markers such as MELD and PELD have been developed to assess the degree of disease, mortality, and priority on the liver transplant waiting list in liver patients [22].

The MELD score was developed using a patient's serum bilirubin, creatinine, and INR. It is a scoring system that aims to predict the severity of chronic liver disease and survival in patients with acute liver failure [23-27]. We could not find any study in the literature that evaluated the relationship between haematological parameters and mortality scores in patients with WD. However, in a study of 91 patients with non-acetaminophen-related acute liver failure, the MELD score was reported to have similar sensitivity and specificity to the King's College Criteria in predicting mortality (King's College Criteria sensitivity of 88% and specificity of 71%; sensitivity of 79% and specificity of 71% when MELD score ≥32) [24]. In our study, we found a statistically significant positive correlation between WBC, Menzer index, MCV, and RDW values and MELD-PELD scores. In addition, there was a significant negative correlation between Hb, RBC, PLT, PCT, and Hb/RDW ratio values and MELD and PELD scores (Table 2). These haematological parameters showed sensitivity and specificity close to the literature in detecting the presence of cirrhosis in patients. We think that these haematological differences developing in cirrhotic WD may be due to the effects of accompanying portal hypertension and organ sizes.

It has been emphasised that noninvasive biochemical scores (FIB 4, APRI) can be used to evaluate the development of cirrhosis and liver fibrosis in chronic liver disease, which can be an alternative to invasive diagnostic methods [28]. In our study, the ability of scores used to evaluate liver fibrosis, such as FIB4, APRI, and Metavir fibrosis score, to predict cirrhosis in Wilson patients was also evaluated. We found that there was no correlation between these scores evaluating liver fibrosis and haematological parameters and that FIB4 and APRI scores did not constitute a significant cut-off point in determining cirrhosis (AUC: 521, p = 0.872; AUC: 0.526, p = 0.702).

The retrospective nature of our study leads to uncertainty in the preliminary preparation stages that may affect haematological and biochemical analyses. Since the biochemical parameters were not always collected under standardised conditions, such as a fasting state or at the same time of day, circadian variations might have influenced the results. Moreover, information about concomitant medications or dietary factors that could potentially alter laboratory values was not available in some patients. These factors may have contributed to minor variability in the analysed parameters.

## Conclusions

The data obtained in our study provide important information that will assist clinicians in predicting cirrhosis in paediatric WD patients. The significant correlation between haematological parameters and prognostic scores suggests that they may have an impact on prognosis, but this should be supported by further prospective studies.

## References

[1] Dzieżyc K, Litwin T, Członkowska A (2017). Other organ involvement and clinical aspects of Wilson disease. Handbook of Clinical Neurology.

[2] Schilsky ML (2014). Wilson disease: clinical manifestations, diagnosis, and treatment. Clin Liver Dis (Hoboken).

[3] Walabh P, Meyer A, de Maayer T, Moshesh PN, Hassan IE, Walabh P, Hajinicolaou C (2022). Prognostic factors and scoring systems associated with outcome in pediatric acute liver failure. BMC Pediatr.

[4] Güngör Ş, Selimoğlu MA, Bağ HG, Varol FI (2020). Is it possible to diagnose fulminant Wilson's disease with simple laboratory tests?. Liver Int.

[5] Ferenci P, Caca K, Loudianos G (2003). Diagnosis and phenotypic classification of Wilson disease. Liver Int.

[6] Chen B, Ye B, Zhang J, Ying L, Chen Y (2013). RDW to platelet ratio: a novel noninvasive index for predicting hepatic fibrosis and cirrhosis in chronic hepatitis B. PLoS One.

[7] Wai CT, Greenson JK, Fontana RJ, Kalbfleisch JD, Marrero JA, Conjeevaram HS, Lok AS (2003). A simple noninvasive index can predict both significant fibrosis and cirrhosis in patients with chronic hepatitis C. Hepatology.

[8] Vallet-Pichard A, Mallet V, Nalpas B (2007). FIB-4: an inexpensive and accurate marker of fibrosis in HCV infection. Comparison with liver biopsy and FibroTest. Hepatology.

[9] (2025). MDCalc. https://www.mdcalc.com.

[10] Grudeva-Popova Grudeva-Popova, JG JG, Spasova MI, Chepileva KG, Zaprianov ZH (2000). Acute hemolytic anemia as an initial clinical manifestation of Wilson’s disease. Folia Med.

[11] Steindl P, Ferenci P, Dienes HP (1997). Wilson's disease in patients presenting with liver disease: a diagnostic challenge. Gastroenterology.

[12] Sharma S, Toppo A, Rath B, Harbhajanka A, Lalita Jyotsna P (2010). Hemolytic anemia as a presenting feature of Wilson’s disease: a case report. Indian J Hematol Blood Transfus.

[13] Chen K, Wan Y, Mao J, Lai Y, Zhuo-Ma G, Hong P (2022). Liver cirrhosis prediction for patients with Wilson disease based on machine learning: a case-control study from southwest China. Eur J Gastroenterol Hepatol.

[14] Zhu M, Han M, Xiao X, Lu S, Guan Z, Song Y, Liu C (2019). Dynamic differences of red cell distribution width levels contribute to the differential diagnosis of hepatitis B virus-related chronic liver diseases: a case-control study. Int J Med Sci.

[15] Wang J, Huang R, Yan X (2020). Red blood cell distribution width: a promising index for evaluating the severity and long-term prognosis of hepatitis B virus-related diseases. Dig Liver Dis.

[16] Yu Z, Zhang T, Shen J (2022). Low hemoglobin-to-red cell distribution width ratio is associated with mortality in patients with HBV-related decompensated cirrhosis. Biomed Res Int.

[17] Ramzy I, Fouad R, Salama R (2021). Evaluation of red cell distribution width to platelet ratio as a novel non-invasive index for predicting hepatic fibrosis in patients with chronic hepatitis C. Arab J Gastroenterol.

[18] Huang A, Chang B, Sun Y, Lin H, Li B, Teng G, Zou ZS (2017). Disease spectrum of alcoholic liver disease in Beijing 302 Hospital from 2002 to 2013: a large tertiary referral hospital experience from 7422 patients. Medicine (Baltimore).

[19] Yang J, Yan B, Yang L (2018). Macrocytic anemia is associated with the severity of liver impairment in patients with hepatitis B virus-related decompensated cirrhosis: a retrospective cross-sectional study. BMC Gastroenterol.

[20] Mangal AK, Sharma D, Kumar P, Nagar GL (2023). To study mean corpuscular volume in alcoholic liver disease patients and to compare with Child-Pugh score to predict the severity. J Assoc Physicians India.

[21] Ustaoglu M, Aktas G, Avcioglu U, Bas B, Bahceci BK (2021). Elevated platelet distribution width and red cell distribution width are associated with autoimmune liver diseases. Eur J Gastroenterol Hepatol.

[22] Raza MH, Kwon Y, Kobierski P (2023). Model for end-stage liver disease/pediatric end-stage liver disease exception policy and outcomes in pediatric patients with hepatopulmonary syndrome requiring liver transplantation. Liver Transpl.

[23] Yantorno SE, Kremers WK, Ruf AE, Trentadue JJ, Podestá LG, Villamil FG (2007). MELD is superior to King's College and Clichy's criteria to assess prognosis in fulminant hepatic failure. Liver Transpl.

[24] Parkash O, Mumtaz K, Hamid S, Shah SH, Jafri W (2012). MELD score: utility and comparison with King's College criteria in non-acetaminophen acute liver failure. J Coll Physicians Surg Pak.

[25] Katoonizadeh A, Decaestecker J, Wilmer A (2007). MELD score to predict outcome in adult patients with non-acetaminophen-induced acute liver failure. Liver Int.

[26] Zaman MB, Hoti E, Qasim A (2006). MELD score as a prognostic model for listing acute liver failure patients for liver transplantation. Transplant Proc.

[27] Schmidt LE, Larsen FS (2007). MELD score as a predictor of liver failure and death in patients with acetaminophen-induced liver injury. Hepatology.

[28] Pastrovic F, Madir A, Podrug K (2022). Use of biochemical parameters for non-invasive screening of oesophageal varices in comparison to elastography-based approach in patients with compensated advanced chronic liver disease. Biochem Med (Zagreb).

